# Access to health insurance coverage among sub-Saharan African migrants living in France: Results of the ANRS-PARCOURS study

**DOI:** 10.1371/journal.pone.0192916

**Published:** 2018-02-15

**Authors:** Nicolas Vignier, Annabel Desgrées du Loû, Julie Pannetier, Andrainolo Ravalihasy, Anne Gosselin, France Lert, Nathalie Lydié, Olivier Bouchaud, Rosemary Dray Spira, the PARCOURS Study Group

**Affiliations:** 1 INSERM, Sorbonne Université, Institut Pierre Louis d’Épidémiologie et de Santé Publique (IPLESP), Department of Social Epidemiology, Paris, France; 2 Groupe hospitalier Sud Ile-de-France, Department of Infectious and Tropical Diseases, Melun, France; 3 Sorbonne Paris Cités, IRD, CEPED, ERL INSERM 1244 SAGESUD, Paris, France; 4 INSERM, Centre for Research in Epidemiology and Population Health (CESP-U 1018), Villejuif, France; 5 Santé Publique France, French National Agency of Public Health, Saint-Maurice, France; 6 Paris 13 University, Avicenne Hospital, Assistance Publique-Hôpitaux de Paris (AP-HP), Department of Infectious and Tropical diseases, and Laboratoire Educations et Pratiques de Santé (LEPS EA 3412), Bobigny, France; Rutgers School of Public Health, UNITED STATES

## Abstract

**Background:**

Migrants’ access to care depends on their health insurance coverage in the host country. We aimed to evaluate in France the dynamic and the determinants of health insurance coverage acquisition among sub-Saharan migrants.

**Methods:**

In the PARCOURS life-event retrospective survey conducted in 2012–2013 in health-care facilities in the Paris region, data on health insurance coverage (HIC) each year since arrival in France has been collected among three groups of sub-Saharan migrants recruited in primary care centres (N = 763), centres for HIV care (N = 923) and for chronic hepatitis B care (N = 778). Year to year, the determinants of the acquisition and lapse of HIC were analysed with mixed-effects logistic regression models.

**Results:**

In the year of arrival, 63.4% of women and 55.3% of men obtained HIC. But three years after arrival, still 14% of women and 19% of men had not obtained HIC. HIC acquisition was accelerated in case of HIV or hepatitis B infection, for migrants arrived after 2000, and for women in case of pregnancy and when they were studying. Conversely, it was slowed down in case of lack of a residency permit and lack of financial resources for men. In addition, women and men without residency permits were more likely to have lost HIC when they had one.

**Conclusion:**

In France, the health insurance system aiming at protecting all, including undocumented migrants, leads to a prompt access to HIC for migrants from sub-Saharan Africa. Nevertheless, this access may be impaired by administrative and social insecurities.

## Introduction

With 244 million international migrants worldwide and increasing migration to Europe, migration is a global phenomenon that could influence the health of individuals [1, 2]. The question of the health of migrants and their access to the health care system is therefore more acute. Despite an increasing focus on migration globally, there are insufficient data on the interaction between migration and health and of how health systems cope with immigration [3]. The migrant population is very heterogeneous, depending on their country of origin, the circumstances of migration and the living condition at arrival in the host country. However, many migrants arriving in Europe from developing countries, and particularly those arriving from Africa, experience difficult migration pathways and find themselves in a precarious situation after arrival in the host countries [4]. They are thus considered at higher risk for a range of health problems in Europe, especially the undocumented ones, which are the most vulnerable [5, 6]. This higher risk is partly due to poor socioeconomic conditions and, in some countries, is due to the lack of rights to health coverage for undocumented migrants [7–9]. Existing evidence from different European countries highlights the difficulties to access health services that migrants are facing [10–13]. These difficulties are due to various reasons as lack of health insurance coverage or insufficient knowledge of rights and structures [14–18]. Access to health insurance that provides coverage for medical and hospital care is a major determinant of healthcare access and reduction of morbidity and mortality [19–22]. Universal health coverage is the subject of a globally approved United Nations General Assembly resolution and is the third Sustainable Development Goal of the UN Development Programme [23, 24]. In addition, the specific challenges encountered in the field of migration and health has been recognized as a priority for research, as the need for better evidence to improve health system responses to migration [3, 25].

In France, the health-care system was built at the end of World War II as part of the social security system and, to date, has continuously improved to ensure health access for all [26]. It is based on a public health insurance system named Health Insurance (HI) (see the supporting information S1 for a detailed description) (S1 Text). HI is based on compulsory social insurance funded by social contributions. Government provides basic Health Insurance Coverage (HIC) for French and foreign people residing in France regularly and working, studying or being linked to a recipient of the social security system (assignee). This Standard health Insurance is supplemented by a voluntary private insurance that covers health care costs not reimbursed. However, such supplementary insurance is less common among the lower segments of the population. In 1999 was created the Universal Health insurance Coverage (UHC) for French people and foreign nationals living legally in France under an income ceiling and who were previously excluded from Health Insurance based on administrative and/or socio-professional criteria. UHC provided them the right to basic health insurance for basic welfare and, depending on income, for complementary health insurance. Thus, UHC is basic health insurance coverage for inactive people living regularly in France without an assignee.

At the same time, the State Medical Assistance (SMA) was created for undocumented immigrants [27]. SMA covers the entire cost of care. Several supporting documents are required to apply: passport or identity card, an address of domiciliation and over three months’ presence in France. Beneficiaries must be below a resource threshold similar to the supplementary UHC coverage threshold (in the order of $ 10,000 annually for a single person). Dependent people can also benefit from State Medical Assistance (i.e. partners and children). In theory, all healthcare professionals are obliged to accept SMA beneficiaries and forbid them from exceeding fee. The period of entitlement is one renewable year. With the SMA, France is one of the few European countries to ensure a wide access to care for undocumented migrants, but through a separate system.[28].

In contrast to these theoretical possibilities of universal access to care, some reports shows that this access to care is not as easy as it should be [20, 26, 29–31]. There is limited empirical research available that analyses migrants’ access to Health Insurance Coverage.

People from sub-Saharan Africa are at a higher risk of HIV and chronic hepatitis B (CHB) infections and need preventive services and access to diagnostic, care and treatment [32, 33]. For migrants living with HIV or CHB, being engaged in care promotes medication adherence, prevents complications, and decreases the risk of transmission [34–36]. Health insurance coverage could play an important role in their diagnosis, entry and retention in care [37].

Using the data from a large life-event survey of people from sub-Saharan Africa living in France with or without HIV or CHB, we aimed to investigate the acquisition time of Health insurance after arrival in France and how acquisition and disruption are associated with social, administrative and medical determinants.

## Methods

### Study design and participants

The PARCOURS study was conducted to analyse how health trajectories and social and migratory paths are interlaced for migrants from sub-Saharan Africa who are living in France. This retrospective quantitative life-event survey was conducted from February 2012 to May 2013 in health-care facilities in the Paris metropolitan area (Ile-de-France). Three groups of migrants born in sub-Saharan Africa have been studied: one group followed in care for HIV infection in dedicated HIV centres (HIV group), one group in care for Chronic Hepatitis B (without concomitant HIV infection) followed in dedicated CHB centres (CHB group), and a third group of people who visited primary-care centres for any reason (reference group). The study used time-location sampling [38], in which healthcare facilities were randomly selected from three exhaustive lists of primary-care centres (including primary-care centres for vulnerable populations), HIV outpatient hospital clinics and hepatitis treatment clinics. We constructed three distinct sampling frames (one for each healthcare specialty) by each half-day that the healthcare facilities were open. All eligible patient visits were included from each healthcare facility and each half-day time interval. To construct a sample that reflected the contribution of the various types of healthcare facility found in Île-de-France, the number of individuals to include from each facility was determined according to the group’s weight within the total population of migrants from sub-Saharan Africa in the Paris metropolitan era. The data were weighted according to each individual’s probability of inclusion in the survey.

Patients were eligible if they were born in sub-Saharan Africa, were citizens of a sub-Saharan African country at birth, were between 18 and 59 years old, and had not been diagnosed with HIV or hepatitis B (for the primary-care group) or with HIV infection or CHB (the other two groups) for at least 3 months. Recruitment occurred at the healthcare facilities. Physicians asked their eligible patients to participate and acquired their written consent.

A trained interviewer administered a face-to-face standardized life-event history questionnaire to each participant. Information collected included sociodemographic characteristics, conditions of migration and life in France, relational, sexual, and reproductive histories, and healthcare pathways that included HIV and hepatitis B virus (HBV) testing, healthcare insurance coverage, and engagement in care. Each parameter of interest was documented year to year from birth until the time of data collection. To collect retrospectively this life-event information, we used the life history calendars or “Ageven” sheet (also known as life event or life grid calendars). This tool has been shown to be effective in reducing recall bias and improving data quality in retrospective studies by providing a graphical time line that helps participants to anchor their responses in relation to different life stages and events.[39–42]

Clinical and laboratory information was documented from medical records. All information was anonymously collected.

The complete survey protocol is registered and available on Clinicaltrials.gov (NCT02566148 https://clinicaltrials.gov/ct2/show/NCT02566148). Other aspects of the study have already been presented elsewhere [43, 44].

### Ethical considerations

The Advisory Committee on Data Collection in Health Research (CCTIRS) and the French Data Protection Authority (CNIL) approved the study protocol (CD-2011-484 approval on 7 December 2011). All information was anonymously collected.

To take into account difficulties in participating in the survey due to poor or no knowledge of the French language, the patient questionnaire was available in French or English, and, by appointment, an interpreter could be made available to conduct the interview in an African language spoken by the respondent.

### Outcomes and variables of interest

For each year between the arrival in France and the year of data collection, HIC was documented. The first outcome was the delay of acquisition of first HIC since arrival in France. HIC was defined as any type of basic HIC that lasted for at least one year without considering supplementary health insurance. The others outcome were incidence of first HIC interruption after obtaining it and basic HIC at the time of the study (HI, UHC, SMA or none).

The fixed covariates for the analysis of the factors associated with the acquisition delay included the period of arrival, the age, the level of education, place of birth and the reported reasons for migration. Living conditions in France were documented for each year between arrival and the year of data collection through several time-dependent variables: permit of residence, housing situation, economic resources, and activity. Medical conditions including pregnancy, hospitalization, HIV and/or CHB diagnosis were dated and treated as time-dependent variables.

### Statistical analyses

The analysis focused on people who arrived in France after 1980, who have been in France at time of interview for at least 2 years, aged over 18 on arrival and without missing data in the model variables. Persons who arrived before 1980 or who were under 18 years of age on arrival were not included. Persons who arrived in the previous year did not allow for a satisfactory analysis of the factors related to time. The database and analysis file for reproducing this analysis is available in supporting information (S1 Table and S2 Text)

Sociodemographic characteristics, including the main reasons for coming to France and the hardships experienced in France were compared between groups with a design-based [chi]2 test to compare proportions. Medians of duration were compared with non-parametric equality-of-medians tests.

Characteristics associated with the acquisition of HIC each year since the time of arrival in France were identified using mixed-effect logistic regression models. Models included both fixed and time-dependent covariates and were systematically adjusted for time since arrival in France.

Given the retrospective nature of the data and the heterogeneity regarding the time since arrival in France, migrants with a delayed access to HIC may have been particularly underrepresented among those who arrived within the most recent period. To assess possible bias, an additional analysis was performed in a database restricted to participants who had been in France for at least 3 years at the time of interview.

In the same way, we analysed factors associated with the loss over time of this first HIC among men and women in the 4 years after it was obtained.

Data were weighted according to each individual’s probability of inclusion in the survey and the weights were applied to all percentages. All analyses were stratified by sex due to differentiated migratory patterns and exchanges with the healthcare system.

All analyses were performed in Stata SE 13.1 (Stata Corporation, College Station, TX, USA).

## Results

### Study population

A total of 1184 (reference group), 1829 (HIV) and 1169 (CHB) individuals met the eligibility criteria, among which 124, 141 and 25, respectively, were not offered participation by their physicians due to health problems or cognitive impairment. Eventually, 763 migrants in the reference group, 926 migrants with HIV, and 778 migrants with CHB agreed to participate. A total of 552 subjects were excluded for different reasons: 76 people arrived in France before 1980, 81 had been in France for less than a year, 210 were under the age of 18 at the time of their arrival, and 185 were excluded because of missing data in the variables. Consequently, a total of 1008 men and 907 women were included in the analysis: 547 in the reference group, 749 in the HIV group and 619 in the CHB group.

The sociodemographic characteristics of the participants are described in Table 1. Women accounted for 55.6% of the reference group, 62.4% of the HIV group, and 26.8% of the CHB group. The median age at arrival was 29 years in the reference group for both sexes. Men and women in the HIV group arrived when they were older. Most came from Western and Central Africa. Men most often reported coming to France to seek work and women reported that they came for family unification. The median duration of residence in France was 9 years [IQR: 2–15] for men and women in the reference group. Absences of residency permits, of personal housing, or of resources on arrival in France were frequent. The absence of a residency permit on arrival was more common in the HIV or CHB groups (Table 1).

**Table 1:** Socio-demographic characteristics by sex and by study group—ANRS PARCOURS study.

	Men				Women			
	Ref. group	HIV	CHB		Ref. group	HIV	CHB	
	N = 265	N = 285	N = 458		N = 282	N = 464	N = 161	
	%	%	%	P value	%	%	%	P value
**Age at arrival in France (years)**								
18–24	27.7	16.6	25.5	0.004	30.5	21.7	25.4	0.06
25–29	34.7	30.1	35.9		32.3	32.5	39.0	
30–60	37.6	53.3	38.6		36.6	45.7	35.6	
**Arrival period in France**								
1980–1999	35.5	42.4	27.3	0.008	39.8	33.2	19.4	0.03
2000–2012	64.5	57.6	72.7		60.3	66.8	80.6	
**Educational level at arrival**								
None/primary	32.9	24.3	38.4	0.08	25.2	24.3	28.3	0.83
Secondary	45.3	48.1	38.9		56.5	59.0	54.5	
Postsecondary	21.8	27.8	22.7		18.3	16.7	17.3	
**Region of birth**								
West Africa	64.8	58.3	81.5	<0.001	53.4	48.8	74.5	<0.001
Central Africa	31.4	39.6	17.1		43.4	48.0	23.2	
East/Southern Africa	0.0	2.0	0.0		3.2	3.2	2.3	
**Duration of stay in France**								
Median	9	11	9.5	0.02	9	10	7	0.01
IQR	2–15	6–21	4–13		2–16	6–14	3–12	
**Reason for coming to France**								
Find work	44.9	44.9	53.0	0.07	24.2	38.2	28.4	0.007
Threatened in his/her country	28.0	18.9	19.5		20.7	11.2	17.1	
Join a family member or study	24.9	27.4	21.5		40.8	40.9	45.4	
Medical reasons	2.2	8.9	6.1		4.3	9.8	9.1	
**Resident permit at arrival**								
None	48.2	53.4	61.9	0.06	38.0	48.5	46.5	0.008
Temporary resident permit	44.6	38.7	32.9		54.1	44.4	46.0	
Resident permit (10 years)	5.3	3.6	2.8		7.4	6.2	3.6	
French nationality	1.9	4.2	2.5		0.5	0.9	4.0	
**Housing situation at arrival**								
Own housing	16.0	19.1	11.8	0.37	35.0	19.2	22.1	0.02
Housed by family	41.4	39.9	46.0		43.5	50.9	44.5	
Associations, worker’s hostels	14.0	11.1	16.1		3.2	2.6	6.8	
No stable housing	28.6	30.0	26.2		18.4	27.3	26.6	
**Resources at arrival**								
Own work	62.8	69.5	68.5	0.41	40.3	47.8	37.6	0.29
From spouse or family	16.4	15.4	17.8		41.1	35.7	46.4	
Government allowances	13.7	7.9	6.9		8.4	7.6	9.0	
No resource	7.1	7.1	6.8		7.3	9.0	7.0	

Note: Weighted percentages. P value: design-based x2 test comparison of proportion and quantile regression with robust variance estimator for median comparison across groups. CHB, chronic hepatitis B; IQR, interquartile range; Ref. group, reference group.

### Delay to acquisition of first HIC since arrival in France

**Fig 1:**
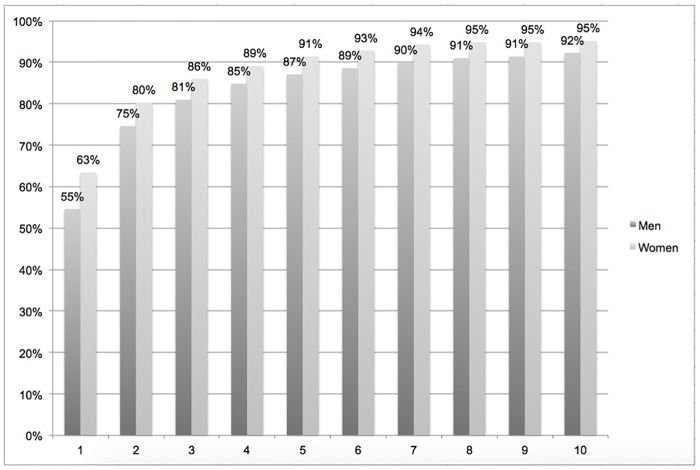
Access to first health insurance coverage by years after arrival in France (N = 1915).

The proportion of participants with HIC is presented year to year after arriving in France in Fig 1. HIC was obtained the year of arrival in France in median (IQR [0–1]) with no difference across groups. Among men, 55.3% had acquired an HIC the first year after arrival. This percentage rises to 74.6% the second year and 80.9% the third year after arrival in France; these figures were 63.4%, 80.2% and 86.0%, respectively, for women.

### Factors associated with acquisition of HIC year to year after arrival in France

In the univariate analysis, women acquired HIC more quickly than men (odds ratio = 1.35, 95% confidence interval [1.13–1.61]). No difference in the access to HIC was observed between groups (OR = 1.10 [0.88–1.37] for HIV Group and OR = 1.11 [0.89–1.38] for CHB group; reference = Ref. group).

Among men, men who arrived after 2000 acquired HIC faster than those who had arrived earlier (Table 2). Men were more likely to acquire HIC during the year of hospitalization and if concerned, once they were diagnosed with HIV or CHB.

**Table 2:** Factors associated with acquisition of health insurance coverage year by year since arrival in France among men and women (mixed-effects logistic regression models).

	Men		Women	
	N = 1008 (2667 PYR)		N = 907 (1916 PYR)	
	Univariate	Multivariate	Univariate	Multivariate
	OR [95% CI]^a^	OR [95% CI]^b^	OR [CI95%]	OR [95% CI]
**Arrival period in France**				
1980–1999	1.00	1.00	1.00	1.00
2000–2012	1.55**[1.25–1.93]	1.57**[1.20–2.06]	1.58**[1.18–2.11]	1.54**[1.12–2.12]
**Age at arrival in France (years)**				
18–24	0.70*[0.54–0.90]	0.76[0.54–1.06]	0.74[0.54–1.03]	0.83[0.58–1.19]
25–29	0.84[0.66–1.08]	1.04[0.76–1.42]	1.04[0.78–1.39]	1.26[0.91–1.74]
30–60	1.00	1.00	1.00	1.00
**Level of education at arrival**				
None/primary	1.00	1.00	1.00	1.00
Secondary	1.10[0.89–1.37]	0.94[0.69–1.27]	1.52*[1.12–2.07]	1.65**[1.21–2.26]
Postsecondary	1.34 [0.96–1.86]	0.62*[0.43–0.92]	2.09**[1.36–3.22]	1.38[0.81–2.36]
**Place of birth**				
West Africa	0.97[0.75–1.27]	/	0.76[0.58–1.00]	1.00[0.74–1.34]
East, central or southern Africa	1.00	/	1.00	1.00
**Reason for coming to France**				
Find work	0.70* [0.50–0.98]	0.77 [0.55–1.09]	0.73* [0.55–0.98]	0.79 [0.56–1.12]
Medical reasons	1.39 [0.86–2.26]	0.97 [0.53–1.77]	1.19 [0.74–1.94]	1.22 [0.67–2.23]
Threatened in your country	1.09 [0.74–1.59]	0.88 [0.56–1.37]	0.86 [0.55–1.36]	0.75 [0.45–1.25]
Join a family member or study	1.00	1.00	1.00	1.00
**Permit of residence**°				
No residency permit	0.38*[0.18–0.77]	0.36**[0.18–0.72]	0.46*[0.23–0.90]	0.44*[0.24–0.80]
Temporary residence permit	1.66 [0.77–3.56]	1.87 [0.92–3.82]	2.25*[1.12–4.50]	1.83 [0.98–3.44]
Residence permit (10 years)	1.00	1.00	1.00	1.00
French nationality	3.00 [0.98–9.21]	3.15 [0.99–10.03]	4.57* [1.45–14.38]	4.84* [1.23–19.13]
**Housing situation**°				
Own housing	1.00	1.00	1.00	1.00
Housed by your family	0.74 [0.54–1.03]	0.83 [0.59–1.17]	0.68*[047–0.97]	0.89 [0.60–1.31]
Associations	1.38 [0.50–3.85]	2.14 [0.85–5.40]	1.10 [0.49–2.49]	0.78 [0.30–2.01]
No stable housing	0.95 [0.67–1.34]	0.95 [0.65–1.38]	0.75 [0.50–1.10]	0.87 [0.57–1.33]
Migrants workers’ homes	0.70 [0.48–1.01]	0.95 [0.59–1.54]	1.54 [0.59–4.03]	1.67 [0.54–5.18]
**Resources**°				
Own work	1.00	1.00	1.00	1.00
From spouse or family	1.01 [0.77–1.34]	1.39 [0.90–2.15]	1.06 [0.78–1.45]	0.86 [0.56–1.33]
Government allowances	2.36**[1.36–4.11]	1.87 [0.91–3.84]	1.80 [0.99–3.26]	0.98 [0.53–1.79]
No resource	0.56*[0.38–0.84]	0.52*[0.29–0.96]	0.83 [0.54–1.29]	0.55 [0.30–1.03]
**Occupation**°				
Worker	1.00	1.00	1.00	1.00
Inactive	0.89 [0.69–1.14]	0.68 [0.46–1.02]	1.27 [0.96–1.69]	1.31 [0.88–1.94]
Student	1.61 [0.89–2.93]	0.88 [0.47–1.67]	15.96**[7.64–33.33]	12.47**[5.17–30.07]
**Pregnancy**° **(of the partner for men)**				
Yes	1.43 [0.96–2.12]	1.23 [0.78–1.94]	2.13**[1.45–3.12]	2.04**[1.35–3.07]
No	1.00	1.00	1.00	1.00
**Hospitalization**°				
Yes	3.48**[1.83–6.62]	1.77 [0.78–4.00]	4.99*[1.07–23.24]	1.24 [0.08–18.99]
No	1.00	1.00	1.00	1.00
**Diagnosed for HIV**°				
Yes	2.09**[1.43–3.05]	1.72*[1.10–2.69]	3.31**[2.28–4,80]	2.75**[1.77–4.25]
No	1.00	1.00	1.00	1.00
**Diagnosed for CHB**°				
Yes	3.33**[2.36–4.71]	3.17**[2.17–4.64]	3.03**[1.78–5.16]	2.63**[1.42–4.87]
No	1.00	1.00	1.00	1.00

Note: PYR: person-years at risk; CI, confidence interval;

*P < 0.05.

**P < 0.01.

^a^Odds ratio taking into account time.

^b^Odds ratio taking into account time and adjusted for covariate.

°Time-varying variables.

Conversely, men were less likely to obtain HIC during years without a residency permit and during years without resources.

In the multivariate analysis, characteristics that were significantly associated with a faster access to HIC were the arrival in France after the year 2000 (adjusted OR = 1.57 [1.20–2.06]) and HIV or CHB diagnosis (aOR = 1.72 [1.10–2.69] and 3.17 [2.17–4.64]). Characteristics associated with a delayed access to HIC were the absence of a residency permit (aOR = 0.36 [0.18–0.72]) and the absence of resources (aOR = 0.52 [0.29–0.96]).

Among women, the same associations were observed with arrival times after 2000, hospitalization, being diagnosed with HIV or CHB and lack of a residency permit (Table 2). In addition, women were more likely to have acquired HIC when they had a secondary level of education or higher at arrival. Furthermore, women were more likely to acquire HIC during the year of a pregnancy and during the years that they were students.

In the multivariate analysis, characteristics that were significantly associated with a faster access to HIC were arrival in France after the year 2000 (aOR = 1.54 [1.12–2.12]), secondary level of education at arrival (aOR = 1.65 [1.21–2.26]), French nationality (aOR = 4.84 [1.23–19.13]), years of school (aOR = 12.47 [5.17–30.07]), year of a pregnancy (aOR = 2.04 [1.35–3.07]) and HIV or CHB diagnosis if concerned (aOR = 2.75 [1.77–4.25] and 2.63 [1.42–4.87]]). The only characteristic associated with a delayed access to HIC was the absence of a residency permit (aOR = 0.44 [0.24–0.80]).

When the same analysis was performed on the restricted database (first 3 years after the arrival in France in people who had arrived more than 3 years ago, N = 1736), the period effect was still significant. Participants who arrived after the year 2000 acquired HIC more rapidly than participants who had arrived before the year 2000 (adjusted OR = 1.57 [1.12–2.20] for men and 1.94 [1.38–2.72] for women, detailed results not shown and available on request).

### HIC interruptions

**Fig 2:**
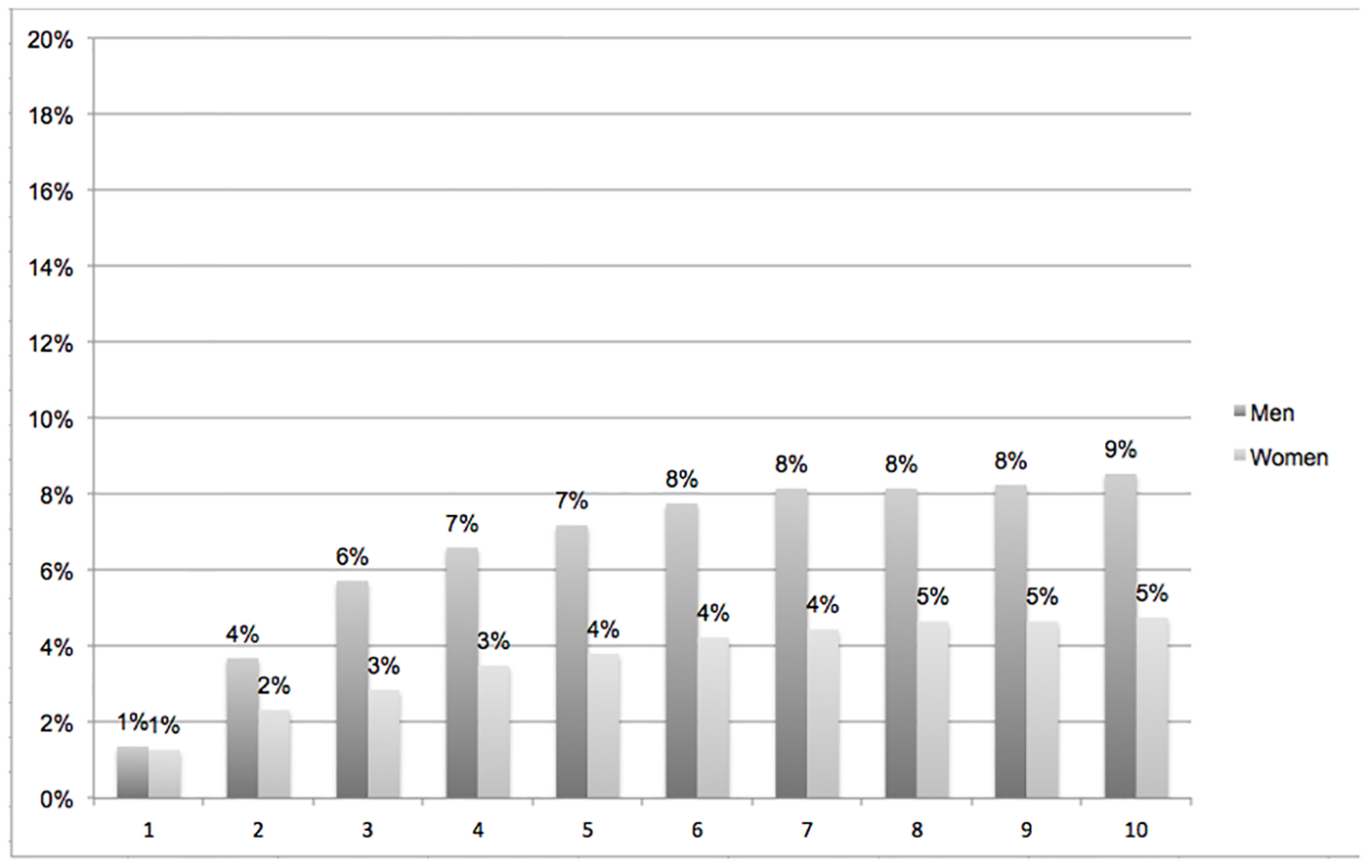
First health insurance coverage interruption by years after obtaining it (cumulative percentage, N = 1915).

Four years after obtaining their first HIC, 7% of men and 3% of women had lost their HIC for more than a year (Fig 2).

Factors associated with HIC interruption year to year in the 4 years after obtaining it among men and women are presented in Table 3. In the univariate analysis, men and women without a residency permit were more likely to have lost HIC. Men who came to France because of a medical reason, men diagnosed with HIV and women under 25 years of age on arrival in France were less at risk of losing their HIC.

**Table 3:** Factors associated with the loss of health insurance coverage year by year in the 4 years after obtaining it among men and women (mixed-effects logistic regression models).

	Men		Women	
	N = 825 (3166 PYR)		N = 762 (3014 PYR)	
	Univariate	Multivariate	Univariate	Multivariate
	OR [95% CI]^a^	OR [95% CI]^b^	OR [CI95%]	OR [95% CI]
**Age at arrival in France (years)**				
18–24	0.82 [0.36–1.85]	/	0.12**[0.02–0.57]	0.14*[0.03–0.64]
25–29	1.59 [0.80–3.17]	/	0.48 [0.14–1.69]	0.54 [0.16–1.77]
30–60	1.00	/	1.00	1.00
**Reason for coming to France**				
Find work	0.97 [0.48–1.97]	0.71 [0.33–1.53]	1.00 [0.24–4.18]	0.51 [0.15–1.81]
Medical reasons	0.12*[0.02–0.97]	0.12 [0.02–1.03]	3.24 [0.52–20.16]	1.14 [0.24–5.45]
Threatened in his/her country	0.42 [0.32–2.24]	0.87 [0.32–2.35]	5.28*[1.37–20.39]	3.08 [0.91–10.41]
Join a family member or study	1.00	1.00	1.00	1.00
**Residence permit**°				
Lack or loss of the resident permit	4.36**[2.20–8.63]	4.33**[2.10–8,92]	5.44**[1.55–19.11]	5.32**[1.68–16.89]
Have a residence permit or French nationality	1.00	1.00	1.00	1.00
**Diagnosed for HIV**°				
Yes	0.34*[0.13–0.88]	0.58 [0.22–1.53]	0.59 [0.20–1.70]	/
No	1.00	1.00	1.00	/
**Diagnosed for CHB**°				
Yes	1.26 [0.62–2.55]	/	1.66 [0.26–10.45]	/
No	1.00	/	1.00	/

Note: PYR: person-years at risk; CI, confidence interval;

*P < 0.05.

**P < 0.01.

^a^Odds ratio taking into account time.

^b^Odds ratio taking into account time and adjusted for covariate.

°Time-varying variables.

In the multivariate analysis, the only characteristic that was significantly associated with interruption in HIC was the lack of a residency permit for both men and women (aOR = 4.51 [2.17–9.37] and 4.41 [1.50–12.94], respectively). Women under 25 years of age on arrival in France lost their HIC less often (aOR = 0.14 [0.03–0.64]).

Of the 84 participants who lost health insurance coverage in the four years after obtaining it, 62% (N = 49) did not have a residency permit during the year of lapsed HIC. Among them, 42% (N = 22) previously had a residency permit and had lost it.

### HIC at the time of the study

At the time of the survey, most African migrants had basic health insurance coverage (from 88.5 to 98.3% depending on the group and sex), most often the Health Insurance (Table 4). The Universal Health insurance Coverage (UHC) was often used (from 17.7 to 24.9%). The State Medical Assistance (SMA) was more frequent in the CHB group (24.0% of men and 21,9% of women) and among men in the reference group (13.6%).

**Table 4:** Basic health insurance coverage at the time of the survey by sex and by study group—ANRS PARCOURS study.

	Men				Women			
	Ref. group	HIV	CHB		Ref. group	HIV	CHB	
	N = 265	N = 285	N = 458		N = 282	N = 464	N = 161	
	%	%	%	P value	%	%	%	P value
Health Insurance (HI)	56.1	68.2	54.5	<0.001	60.6	69.2	54.1	0.002
Universal Health insurance Coverage (UHC)	18.8	20.6	17.7		24.9	22.5	21.1	
State Medical Assistance (SMA)	13.6	7.2	24.0		8.8	6.6	21.9	
None	11.5	4.0	3.8		5.8	1.7	2.9	

Note: Weighted percentages; P value: design-based x2 test comparison of proportion comparison across groups; CHB, chronic hepatitis; Ref. group, reference group.

In the reference group, 11.4% of men and 5.8% of women were uninsured (p = 0.05). This proportion was less frequent in HIV and CHB groups in both sexes (p<0.01). Uninsured participants arrived in France more recently (in median 2 years before vs 10 years for others, p<0.001) and were more often without resident permit (72.2% vs 15.7% of those with a health insurance coverage, p<0.001).

## Discussion

The PARCOURS survey provides original life-event data on documented and undocumented migrants living in France. This study is the first to have evaluated access to Health Insurance Coverage (HIC) among migrants year to year after their arrival in Europe. It shows that migrants from sub-Saharan Africa quickly gain access to HIC after their arrival, especially where they are in a health need. Nevertheless, this access is impaired by administrative and social insecurities.

For most migrants, access to HIC occurs in the first year after arrival in France. This finding emphasizes the positive role played by the existence of French regulations that allow health coverage for all citizens, including undocumented migrants. A comparative study of regulation regarding access to health care for undocumented migrants in European Union had placed France among the countries where access was the highest [45]. Migrants who arrived after the year 2000 were more likely to have acquired HIC early. In France, this could be related to the implementation of the UHC for unemployed persons, including asylum seekers, and of the SMA for undocumented migrants in 1999 [27]. Previously, these people could not benefit from Health Insurance system but only from incomplete social assistance granted by local authorities.

Despite this apparent good access, access to HIC for migrants is not always effective and requires knowledge of the French system and social assistance for access [20, 26, 29–31]. Majority of people who have recently arrived in France are not informed of their rights and existing HIC for undocumented migrants. In the Doctors of the World medical centres, only 14.2% of people who can theoretically benefit from health cover have open rights [29]. They could also be afraid to interact with the institutions for fear to be arrested and held in detention. Among the consultants without residence permit, 35% declare to limit their movements for fear of being arrested and nearly one in four missed an address to access the rights [29]. According to the Platform for International Cooperation on Undocumented Migrants and NGOs, thousands of undocumented migrants in France do not have the SMA coverage to which they are entitled [12, 27, 29, 30, 46]. The main reasons cited include uneven interpretation and implementation of the law across the different social security desks, undocumented migrants’ lack of awareness of the law, lack of acceptable identification documents or adequate evidence regarding residency requirements, language barriers and the fear of being arrested. The defender of rights, that is an independent constitutional authority, has also documented many barriers to rights in France [30]. It noted, in particular, that social security desks sometimes require excessively restrictive entitlement conditions and request for unjustified documents. Institutional barriers to access have also been reported by French NGOs [29, 47]. These practices can thus be interpreted as a way of limiting the goals of the law. One of the reasons put forward by the desks for this practice is the fight against fraud. These administrative practices and abuses are variable in the territory and could be corrected through training and control.

As described before, newly arrived migrants often go through an extended period with hardships (lack of residency permit, economic resources, and housing) in France [4, 43]. Half of the women did not obtain their first valid, one-year residency permit until their third year in France, and half of men obtained this permit in their fourth year. When they obtain a residency permit, it is often a temporary permit that could be not renewed the year after. The Parcours study shows that the absence of a residency permit delayed the acquisition of HIC and was the main reason for HIC lapse. Undocumented migrants cannot benefit SMA in the first three months after their arrival in France and do not always access their rights beyond as has been described above. It is also important to note that the major French surveys addressing the issue of access to care systematically exclude undocumented migrants because of research legislation. This results shows the effect of the management of residence permits with delays and interruptions linked to the immigration policy. The lack of financial resources is also an obstacle to access HIC in men, once again emphasizing the weight of social and administrative insecurity in the access to care. Thus, despite the introduction of UHC and SMA for unemployed and undocumented migrants residing in France, illegal stay and financial hardship remain barriers to access to care.

During this hardship period, health is not a priority and access to the legal rights for medical assistance are often restricted to situations where there is an acute health concern and/or severe illness. Thus, contacts within the health care system, particularly at the time of diagnosis or complication related to HIV or CHB infections, pregnancy and hospitalization, promote health coverage. Due to a need for care, contacts within the health system facilitate access to social assistance and therefore make effective the health coverage rights provided by law. Furthermore, HIV diagnosis allows applying for a residency permit for health reasons. This may play a role in better access to HIC. In addition, there is no restriction to access to care in the case of pregnancy in France.

Among women, the level of education and current student status appear to be factors favouring HIC. This trend was described elsewhere and particularly in another large French study, where education and incomes appear to be the most important drivers of inequalities between French and immigrant populations in the propensity to access a medical specialist [20, 48].

The lack of basic health insurance coverage at the time of the study is more frequent in the reference group, among participants recently arrived and without resident permit. This confirms that despite the right to State Medical Assistance for undocumented migrants in France, some do not apply.

This study is limited by focusing on patients who are engaged in care. The distribution of Health Insurance Coverage for migrants in care may be different from that of persons not in care. Additionally, our findings may not be generalized to all HIV or CHB patients and care settings since it was conducted only in the Paris metropolitan area. However, 60% of sub Saharan migrants in France live in the Paris area and our sample is highly diversified, with patients having a variety of demographic and clinical characteristics.

## Conclusions

In conclusion, the French social security system provides quick access to Health Insurance Coverage for the majority of immigrants arriving in France. This access is facilitated by the existence of the Health Insurance and, since 2000, by the existence of the Universal Health insurance Coverage and the State Medical Assistance. However, despite a system built to facilitate access to care for all, including undocumented migrants, socioeconomic and residency permit insecurity remain as barriers to full access. At the time of questioning of the French social model in the context of the increasing arrival of refugees, vigilance is essential to continue to secure their access to HIC, which is a condition for access to care. In particular, it is a priority to maintain the State Medical Assistance and the complementary Universal Health insurance Coverage, or better to merge it into the Health Insurance. This is all the more important as the benefit of the UHC also concerns, beyond migrants, all people in precarious situations in France. It is also important to develop actions to facilitate access to rights and care for newly arrived migrants. This is of particular interest for migrants living with HIV or CHB to improve early diagnosis, linkage to and retention in care. This is in line with the individual and public health benefits associated with HIV care and treatment: improved health outcomes and reductions in transmission risks. It is also essential to homogenize European policies to achieve the United Nations goal of universal health coverage.

## Supporting information

S1 Text French health protection system. (DOCX)

S2 Text Stata do file. (DOCX)

S1 Table Health coverage database. (XLSX)
