# Supplementary material for: Access to health insurance coverage among sub-Saharan African migrants living in France: Results of the ANRS-PARCOURS study
Source: PLoS One. 2018 Feb 15;13(2):e0192916. doi: 10.1371/journal.pone.0192916 (PMC5814022; doi:10.1371/journal.pone.0192916)
Supplement: S1 Text — (DOCX) [file pone.0192916.s001.docx]

# S1 Text. French health protection system

In France, the health-care system was built at the end of World War II in 1945 as part of the social security system (SS). The French health-care system is based on compulsory social insurance funded by social contributions, co-administered by workers’ and employers’ organizations under State control and driven by highly redistributive financial transfers.

The health insurance system is based on a public health insurance system named Health Insurance (HI). HI provides basic health insurance coverage for French and foreign people residing in France regularly and working, studying or being entitled to a recipient of SS (assignee). The universality of the solidarity-based coverage was enhanced in 1999 with the creation of the Universal basic Health insurance Coverage (UHC) for populations previously excluded from HI. Thus, UHC is a basic health insurance for inactive people living regularly in France without an assignee. In 2000, the State Medical Assistance (SMA) established free health insurance coverage for undocumented immigrants in France for more than 3 months who were below a certain resource threshold. The SMA is based on national legislation (Article L. 251-1 of the Code of Social Action and Families of 23/12/2000 and in Title IV of the Decree of 02/09/1954 (modified 28/07/2005)).

Finally, for foreign people in France for less than 3 months or above the resource threshold, it is possible to use the Urgent and Vital Care System (UVCS) to cover the costs of both urgent care and comprehensive care for pregnant women and children who went to the hospital.

The HI covers only basic parts of the cost of care, which equates to approximately 75% of overall individual health expenditures. A large proportion of the population subscribe to complementary health insurance coverage with paid registration to cover a significant part of the remaining costs. People below a certain resource-ceiling, including beneficiaries of the UHC, are eligible for free Complementary Universal Health Coverage (C-UHC). SMA already covers the supplementary part of care and combines basic and complementary health insurance coverage. In addition, care for 30 chronic diseases, including human immunodeficiency virus (HIV) and chronic hepatitis B infection (CHB) with treatment indications, are 100% supported in France as part of 30 Long Term Condition (*30 LTC*). Prenatal care is also fully covered

Finally, people without any health insurance can be taken care of free of charge in dedicated hospital units called Health care access offices (Permanence d’accès aux soins de santé).

| Types of basic health insurance coverage in France | | |
| --- | --- | --- |
| Health insurance coverage | Term in French | Conditions |
| Health Insurance (HI)* | Assurance maladie | Living regularly in France and working, studying or being an assignee of an HI recipient |
| Universal basic Health insurance Coverage (UHC)* | Couverture maladie universelle | Living regularly for more than 3 months in France and being inactive and not being an assignee of an HI recipient |
| State Medical Assistance (SMA) | Aide Médicale d’Etat | Undocumented migrants living for more than 3 months in France and below a certain resource threshold |
| Urgent and Vital Care System (UVCS) | Dispositif des soins urgents et vitaux | People without health insurance coverage requiring urgent care |
| 30 Long Term Condition | Affections longue durée 30 | Care for one of 30 listed diseases |

*Since 2016, the UHC has been merged with HI

| Types of supplementary Health insurance coverage in France | | |
| --- | --- | --- |
| Health insurance coverage | Term in French | Conditions |
| Private assurance or collective assurance | Assurance privée, mutuelle ou complémentaire santé | Pay a monthly membership |
| Complementary Universal basic Health insurance Coverage (C-UHC) | Couverture maladie universelle complémentaire | Living regularly for more than 3 months in France and being inactive and not being below a certain resource threshold |
| State Medical Assistance (SMA) | Aide médicale d’état | Undocumented migrants living for more than 3 months in France and below a resource threshold |
| 30 Long Term Condition | Affections longue durée 30 | To be followed for one of 30 listed diseases |
