# Supplementary material for: Access to health insurance coverage among sub-Saharan African migrants living in France: Results of the ANRS-PARCOURS study
Source: PLoS One. 2018 Feb 15;13(2):e0192916. doi: 10.1371/journal.pone.0192916 (PMC5814022; doi:10.1371/journal.pone.0192916)
Supplement: S2 Text — (DOCX) [file pone.0192916.s002.docx]

/*Access to Health Insurance Coverage among Sub-Saharan African Migrants Living

in France: Results of the ANRS-PARCOURS Study.*/

keep ID GRP SEXE CLAGE PERIODE_TEMP2 NIV_ET_ARRIV2 REGCL3 REG2 SEJOUR RAISON_ARRIV3 /*

*/ TS_4cl RESID_5cl RESID_ARRIV4CL TYPE_RESS_TEMPS ACTI_MAFE_TEMPS2 GROSSESSE HOSPI DIAG_VIH /*

*/ DIAG_VHB YEAR_Cl PERIODE_COUV_MAL SEJOUR sejour TYPE_COUVMA_3ter ANNEE POIDS_F STRATE PSU

use "$data_s/Base_Health_coverage_Plos_One_1", clear

svyset PSU [pweight=POIDS_F], strata(STRATE) vce(linearized) singleunit(certainty)

/*Socio-demographic characteristics by sex and by study group – ANRS PARCOURS study*/

svy : tab CLAGE GRP if ANNEE==0 & SEXE==1, obs col

svy : tab CLAGE GRP if ANNEE==0 & SEXE==2, obs col

svy : tab PERIODE_TEMP2 GRP if ANNEE==0 & SEXE==1, obs col

svy : tab PERIODE_TEMP2 GRP if ANNEE==0 & SEXE==2, obs col

svy : tab NIV_ET_ARRIV2 GRP if ANNEE==0 & SEXE==1, obs col

svy : tab NIV_ET_ARRIV2 GRP if ANNEE==0 & SEXE==2, obs col

svy : tab REGCL3 GRP if ANNEE==0 & SEXE==1, obs col

svy : tab REGCL3 GRP if ANNEE==0 & SEXE==2, obs col

sum SEJOUR if ANNEE==0 & GRP==3 & SEXE==1, det

sum SEJOUR if ANNEE==0 & GRP==1 & SEXE==1, det

sum SEJOUR if ANNEE==0 & GRP==2 & SEXE==1, det

sum SEJOUR if ANNEE==0 & GRP==3 & SEXE==2, det

sum SEJOUR if ANNEE==0 & GRP==1 & SEXE==2, det

sum SEJOUR if ANNEE==0 & GRP==2 & SEXE==2, det

median SEJOUR if ANNEE==0 & SEXE==1, by (GRP)

median SEJOUR if ANNEE==0 & SEXE==2, by (GRP)

svy : tab RAISON_ARRIV3 GRP if ANNEE==0 & SEXE==1, obs col

svy : tab RAISON_ARRIV3 GRP if ANNEE==0 & SEXE==2, obs col

svy : tab TS_4cl GRP if ANNEE==0 & SEXE==1, obs col

svy : tab TS_4cl GRP if ANNEE==0 & SEXE==2, obs col

svy : tab RESID_ARRIV4CL GRP if ANNEE==0 & SEXE==1, obs col

svy : tab RESID_ARRIV4CL GRP if ANNEE==0 & SEXE==2, obs col

svy : tab TYPE_RESS_TEMPS GRP if ANNEE==0 & SEXE==1, obs col

svy : tab TYPE_RESS_TEMPS GRP if ANNEE==0 & SEXE==2, obs col

/*Analysis: Delay to acquisition of first HIC since arrival in France*/

stset ANNEE [pweight=POIDS_F], id(ID) origin(min)failure (PERIODE_COUV_MAL==1)

*Men

logit PERIODE_COUV_MAL ib3.CLAGE ib1.PERIODE_TEMP2 /*

*/ ib1.NIV_ET_ARRIV2 ib4.RAISON_ARRIV3 /*

*/ ib3.TS_4cl ib1.RESID_5cl ib1.TYPE_RESS_TEMPS ib1.ACTI_MAFE_TEMPS2 ib0.GROSSESSE /*

*/ ib0.HOSPI ib0.DIAG_VIH ib0.DIAG_VHB /*

*/ ib1.YEAR_Cl if SEXE==1 [pweight=POIDS_F],cluster(ID) or

*Women

logit PERIODE_COUV_MAL ib3.CLAGE ib1.PERIODE_TEMP2 /*

*/ ib1.NIV_ET_ARRIV2 ib2.REG2 ib4.RAISON_ARRIV3 /*

*/ ib3.TS_4cl ib1.RESID_5cl ib1.TYPE_RESS_TEMPS ib1.ACTI_MAFE_TEMPS2 ib0.GROSSESSE /*

*/ ib0.HOSPI ib0.DIAG_VIH ib0.DIAG_VHB /*

*/ ib1.YEAR_Cl if SEXE==2 [pweight=POIDS_F],cluster(ID) or

/*Same analysis performed on the restricted database (first 3 years after the arrival

in France in people who had arrived more than 3 years ago)*/

*Men

logit PERIODE_COUV_MAL ib3.CLAGE ib1.PERIODE_TEMP2 /*

*/ ib1.NIV_ET_ARRIV2 ib4.RAISON_ARRIV3 /*

*/ ib3.TS_4cl ib1.RESID_5cl ib1.TYPE_RESS_TEMPS ib1.ACTI_MAFE_TEMPS2 ib0.GROSSESSE /*

*/ ib0.HOSPI ib0.DIAG_VIH ib0.DIAG_VHB /*

*/ ib1.YEAR_Cl if SEXE==1 & ANNEE<=2 & sejour>=3 [pweight=POIDS_F],cluster(ID) or

*Women

logit PERIODE_COUV_MAL ib3.CLAGE ib1.PERIODE_TEMP2 /*

*/ ib1.NIV_ET_ARRIV2 ib2.REG2 ib4.RAISON_ARRIV3 /*

*/ ib3.TS_4cl ib1.RESID_5cl ib1.TYPE_RESS_TEMPS ib1.ACTI_MAFE_TEMPS2 ib0.GROSSESSE /*

*/ ib0.HOSPI ib0.DIAG_VIH ib0.DIAG_VHB /*

*/ ib1.YEAR_Cl if SEXE==2 & ANNEE<=2 & sejour>=3 [pweight=POIDS_F],cluster(ID) or

/*HIC at the time of the study*/

svy : tab TYPE_RESS_TEMPS GRP if ANNEE==0 & SEXE==1, obs col

svy : tab TYPE_RESS_TEMPS GRP if ANNEE==0 & SEXE==2, obs col

save "$data_s/Base_Health_coverage_Plos_One_1", replace

clear
